# Layered Double Hydroxide as a Vehicle to Increase Toxicity of Gallate Ions against Adenocarcinoma Cells

**DOI:** 10.3390/molecules21070928

**Published:** 2016-07-16

**Authors:** Jenny Arratia-Quijada, Selma Rivas-Fuentes, Karina J. Parra Saavedra, Adriana M. Macías Lamas, Gregorio Guadalupe Carbajal Arízaga

**Affiliations:** 1 Departamento de Ciencias de la Salud, Centro Universitario de Tonalá, Universidad de Guadalajara, Avenida Nuevo Periférico 555, 48525 Tonalá, Jalisco, Mexico; jenny.arratia@cutonala.udg.mx; 2 Departamento de Bioquímica, Instituto Nacional de Enfermedades Respiratorias, Calzada de Tlalpan 4502, 14080 Mexico City, Mexico; selma.rivas@iner.gob.mx; 3 Departamento de Química, Universidad de Guadalajara, Marcelino García Barragán 1421, 44430 Guadalajara, Jalisco, Mexico; karina_ps21@hotmail.com; 4 Departamento de Farmacobiología, Universidad de Guadalajara, Marcelino García Barragán 1421, 44430 Guadalajara, Jalisco, Mexico; amaciaslamas@yahoo.com

**Keywords:** nanomedicine, nanocarrier, gallic, nanoparticle

## Abstract

The antineoplasic activity of gallic acid has been reported. This compound induces apoptosis and inhibits the growth of several neoplasic cells. However, this molecule is easily oxidized and degraded in the body. The aim of this work was to intercalate gallate ions into layered double hydroxide (LDH) nanoparticles under controlled conditions to reduce oxidation of gallate and to evaluate its toxicity against the A549 adenocarcinoma cell line. An isopropanol medium under nitrogen atmosphere was adequate to intercalate gallate ions with a lesser oxidation degree as detected by electron spin resonance spectroscopy. Concentrations of the hybrid LDH-gallate nanoparticles between 0.39 and 25 µg/mL reduced the cell viability to 67%, while the value reached with the pure gallic acid and LDH was 90% and 78%, respectively, thus proving that the combination of gallate ions with the inorganic nanoparticles increases the toxicity potential within this dose range.

## 1. Introduction

Layered double hydroxides (LDHs) are synthetic minerals with positively charged brucite-type layers containing divalent and trivalent cations (M^2+^ and M^3+^). A residual electrostatic charge produced by M^3+^ cations is neutralized by anions (A^n-^) intercalated between the layers [1]. The general composition is represented by M^2+^_1-x_M^3+^_x_(OH)_2_(A^n-^)_x/n_•yH_2_O. The proper selection of metal cations allows for the control of biocompatibility; therefore, it is possible to design drug carrier systems [1,2,3]. Those nanoparticles loaded with drugs by adsorption or encapsulation improve the sustained release over a long period of time and provide chemical stability to drug molecules.

On the other hand, gallic acid rises among several natural compounds studied for cancer treatment. This acid can be obtained from a variety of natural products such as gallnut, sumac, black tea and some other plants. It has been demonstrated that this compound possesses antiviral, anti-inflammatory, antimicrobial, antimutagenic and anticarcinogenic activities [4,5,6], and this acid has been used to treat lung, breast, and prostate cancers, as well as leukemia.

As an antioxidant, gallic acid consumes free radicals, thus avoiding damage to nucleic acids or other cell components. Furthermore, gallic acid has the simultaneous ability to act as a pro-oxidant, and this feature induces selective apoptosis in tumor cells [7]. However, this acid is not very stable in plasma and requires an encapsulation system to reduce or avoid degradation.

LDH particles are an alternative medium to provide a longer lifespan to this acid and allow for it to act as vehicle for transportation. To the best of our knowledge, three reports are found in the literature related to hybridization of LDH composed by Mg/Al [8] and Zn/Al cations [4,9] with gallic acid. These papers describe the synthesis and structure of the hybrid products formed and there are no reports related to the toxicity yet. In order to understand the performance of a LDH/gallic system, this report is focused on obtaining a stable product against oxidation during synthesis and evaluating toxicity on lung cancer cells.

## 2. Results and Discussion

### 2.1. Gallate-Intercalated LDH

The solid samples obtained from the three intercalation attempts were fine powders. While the pristine LDH matrix was a white powder, the samples obtained from gallate solutions were strong dark (from water solution under air atmosphere), dark (from isopropanol solution under air atmosphere) and pale green-brown (from isopropanol solution under nitrogen atmosphere).

**Figure 1:**
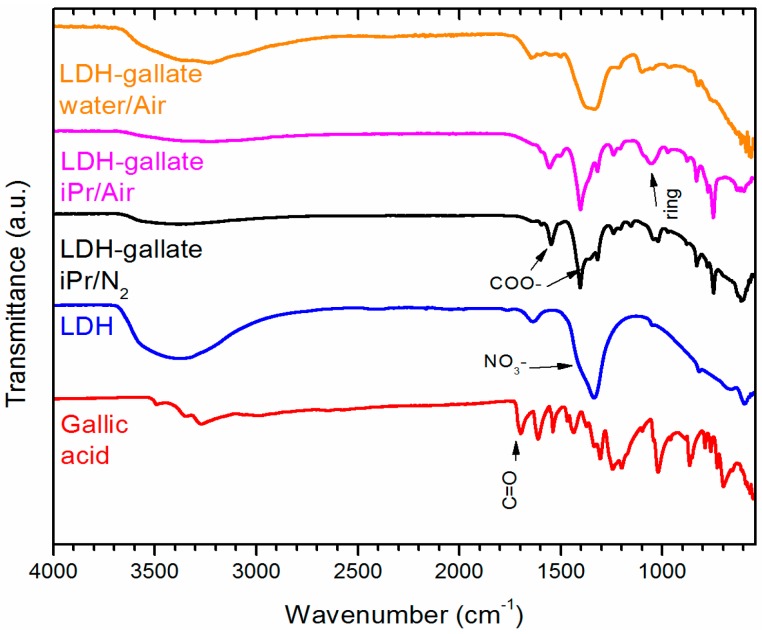
Infrared spectra of gallic acid, the layered double hydroxide (LDH) and the LDH-gallate products obtained from different environments.

The infrared (IR) spectrum of the pristine LDH in Figure 1 presents remarkable bands from -OH, nitrate and M-O stretching modes around 3400, 1370 and 600 cm^−1^, respectively, typical in LDH structures [10]. The set of bands between 1750 and 550 cm^−1^ in the spectra of compounds, synthesized with gallic acid under air atmosphere, correspond to the aromatic ring, phenol and carboxylic groups [4,11].

The product of the reaction conducted in water showed a spectrum with wider bands occurring from a large degree of hydrogen bonding and a probable decomposition of the aromatic ring once the bands at 1541, 1040 and 1355 cm^−1^ were not clearly split. Furthermore, the content of gallate in this sample is lower once the relative intensity of the LDH bands (-OH, NO_3_^−^, and M-O) is larger.

The sample prepared under nitrogen exhibited a spectrum with medium intensity bands at 1541 and 1040 cm^-1^ and is associated to the C=C stretching and O-H in plane deformation [9]; these bands are probably overlapping those of the COO^−^ stretching asymmetric and symmetric modes (see arrows in Figure 1) [9]. A group of bands at 1355 and 1322 cm^−1^ could be produced by nitrate anions (or carbonate if traces of this anion occur) and aromatic O-H bending vibrations [8]. Finally, the C-H deformation of the aromatic ring appearing at 1050 cm^−1^ confirms the gallate presence in the product obtained from the synthesis.

**Figure 2:**
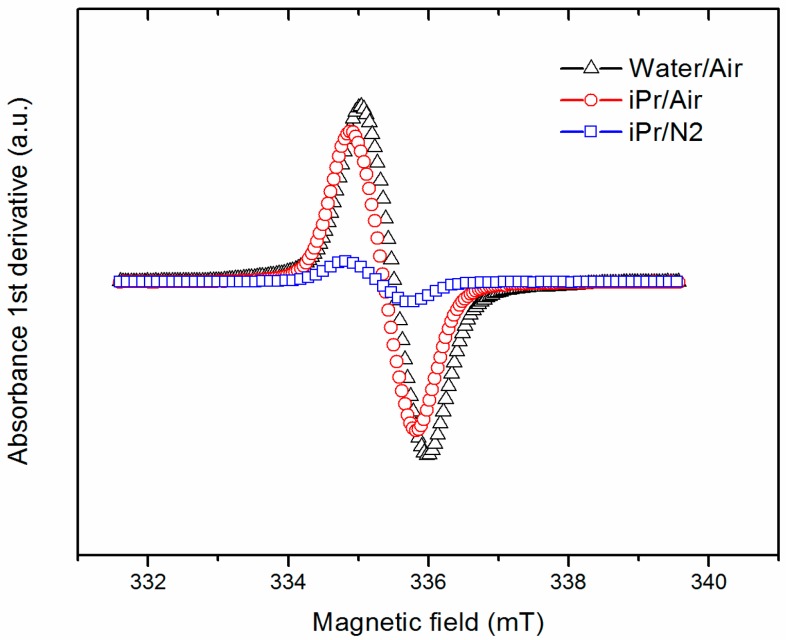
Electron spin resonance (ESR) spectra of the LDH-gallate products obtained from different environments.

A suspicion of degradation/oxidation of gallate ions rose due to the observed dark color of gallate-derivative powders. The analysis by electron spin resonance (ESR) spectroscopy (Figure 2) revealed an absorption signal close to 336 mT, corresponding to organic free radicals (OFR). Considering that the tube was filled with the same amount of sample and was introduced at the same distance for all the samples, the intensity of the signal can be proportionally associated to the OFR content in the gallate-derivative LDH. Thus, the large height in the samples, prepared in water or isopropanol under air, indicates a much larger content of OFR than the sample prepared in isopropanol under nitrogen atmosphere. Published articles reporting gallate-intercalated LDH mention the use of nitrogen atmosphere but do not describe the color as a signal of degradation (related to the formation of free radicals) [4,8]. In this work, the pale-green (red, green, blue color coordinates: 187, 178, 063) sample prepared under nitrogen was selected to conduct the further analyses. After 17 months of storage in a plastic bag and under air atmosphere, the sample synthesized under nitrogen atmosphere presented the same color and the IR spectrum showed the same bands, suggesting that the gallate ions were not altered. Additionally, the comparison of the ESR spectra of this sample with that of the sample prepared in water under air revealed that the relative intensity of signals (I_water/air_/I_iPr/N2_) increased from 8.5 to 12, which could be related to the continuous degradation of gallate in the sample prepared in water/air, while that prepared in isopropanol was more stable.

**Figure 3:**
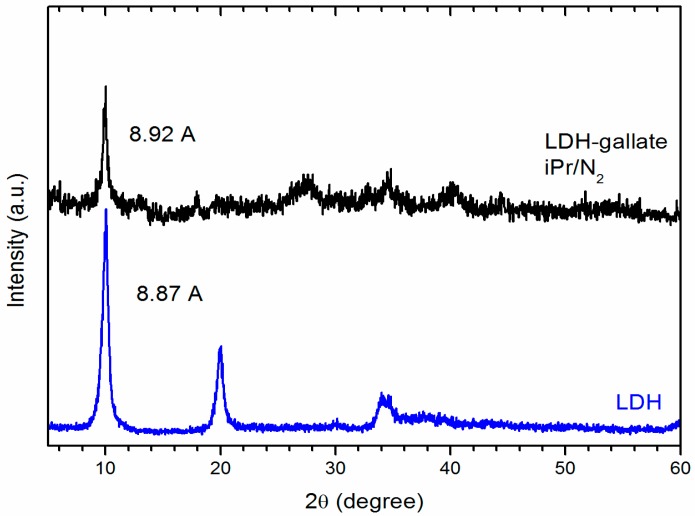
X-ray diffraction (XRD) patterns of the pristine LDH and the LDH-gallate product obtained in isopropanol under nitrogen atmosphere.

X-ray diffraction (XRD) profiles of the pristine LDH and the gallate derivative obtained under nitrogen atmosphere present the most intense reflection at 10 degrees (2θ), which indicates the distance between two contiguous layers that corresponds to 8.87 Å in the pristine matrix and slightly increases to 8.92 Å in the gallate derivative (Figure 3). When gallate ion intercalation occurs, forming a monolayer of anions perpendicularly aligned with the inorganic layers, the basal space increases to 10.1–10.8 Å [8,9]; however, the distance obtained in our product (8.92 Å) could be associated to unexchanged nitrate or gallate ions flattened between LDH layers as proposed in the literature [4].

According to the strong signals from gallate observed in the infrared spectrum, the content of this anion must be significant and this can be explained with the intercalation or adsorption of gallate onto the LDH crystallites [4]. In fact, adsorption is likely occurring as discussed in the transmission electronic microscopy (TEM) section.

**Figure 4:**
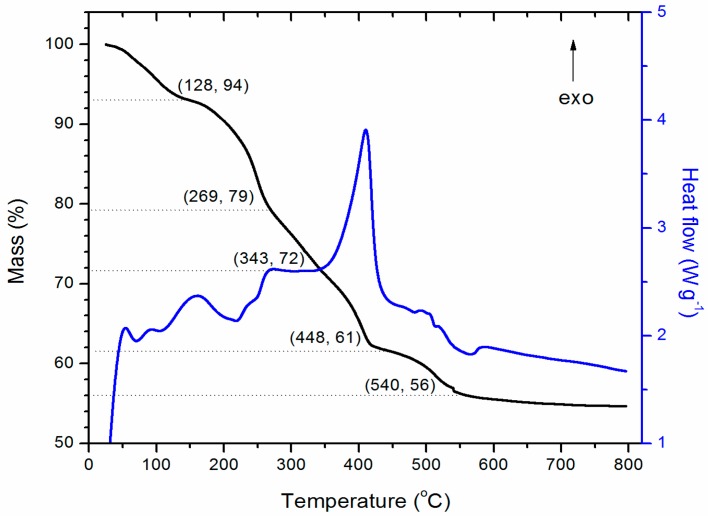
Thermogravimetric analysis and differential scanning calorimetry (TGA and DSC) patterns of the LDH-gallate product obtained in isopropanol under nitrogen atmosphere.

With regards to the thermogravimetric analysis and differential scanning calorimetry (TGA/DSC) data, the profile shown in Figure 4 contains an exothermic peak between 343 and 448 °C which can be exclusively associated to degradation of organic matter in these types of compounds [12,13] and represents a mass loss of 11% (Table 1). For the ideal and total intercalation of gallate, the composition Zn_2.5_Al(OH)_7_(gallate) would contain 24.8% of gallate. According to chromatography results, the content of organic matter was 10.5% in agreement with the loss of organic matter, but this value is far from this theoretical value. However, the theoretical mass of released water produced by hydroxyls is 13.2%, and this is lower than the content in the range between 128 and 343 °C. The presence of nitrate and carbonate ions (as detected by infrared spectroscopy) would explain the decomposition of 22% in this step.

**Figure 5:**
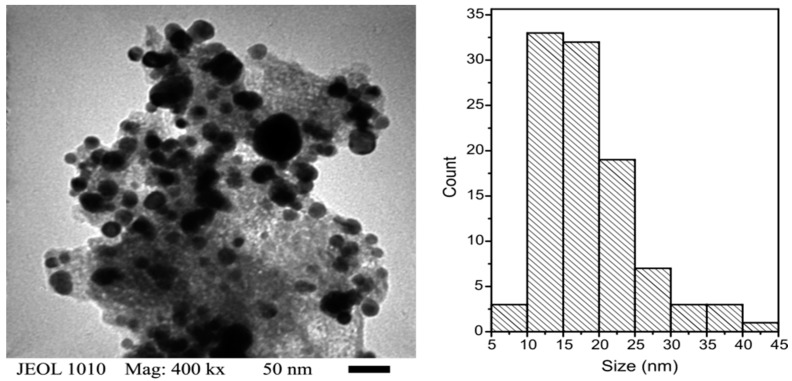
Transmission electron microscopy (TEM) micrograph and size distribution of the LDH-gallate particles.

On the other hand, the TEM micrograph shows particles with the same morphology and a broad size distribution as it is indicated in the histogram (Figure 5), where the mean size is 18.8 nm (S.D. = 6.5). According to the literature, LDH particles with sizes around 50 nm have more capability to cross the membrane of human lung cancer cells in comparison with particles of 100–350 nm [2]; therefore, the size obtained with our method of synthesis is adequate to produce nanometric particles within the range recommended for biological assays [2,14]. However, the particles observed in this analysis are spread along a continuous phase as it occurs in composite materials. The continuous phase must be amorphous since no second phase is detected by XRD, and it could be composed by gallate ions.

Even if gallate were not intercalated within the layered structure, the compound or composite formed is capable of releasing gallate in a sustained manner once the release experiment indicated that 9.8% of gallate is released after 1440 min of agitation in the release experiment. Moreover, if gallate is released at this time, then the hybrid material is stable in the aqueous phase. However, this assay needs further revision since the percentage of released gallate oscillated at some points of the curve and the synthesis of the gallate derivative needs to be improved in order to increase the content of gallate.

### 2.2. Toxicity Assays

**Figure 6:**
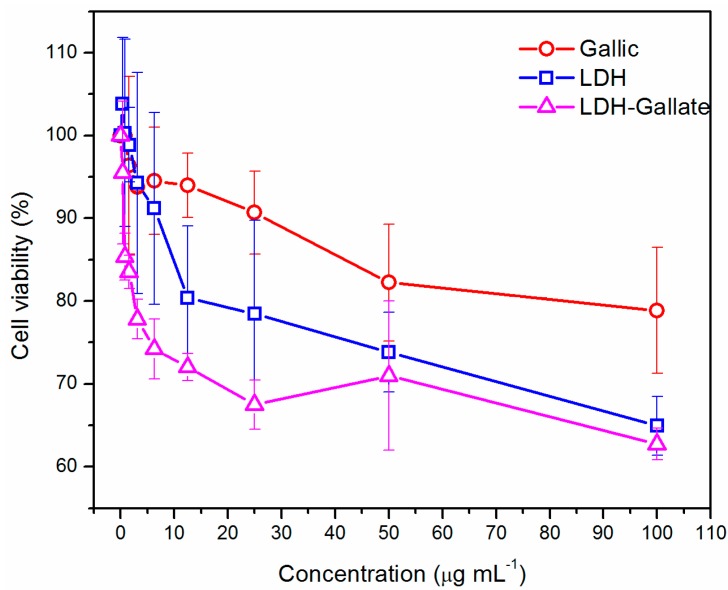
Cell viability of LDH, LDH-gallate nanomaterials and gallic acid on A549 cell line after 24 h of treatment.

Lung cancer cell lines were used to study the possible toxicity and anticancer effectiveness of the LDH and LDH-gallate in a dose-dependent manner. Figure 6 shows the viability of A549 cells after exposure to LDH and LDH-gallate nanoparticles, which is compared with the effect of pure gallic acid after 24 h. At concentrations from 0.39 to 25 µg/mL there is a similar effect with pure gallic acid and the LDH powder. Cell viability decreases as the concentration of both compounds increases. As observed until the point at 25 µg/mL, the mean viability produced by gallic acid and LDH was 90% (5% error) and 78% (11% error), respectively; furthermore, a significant difference between gallic acid and LDH-gallate was found with 12.5 µg/mL; therefore, toxicity is in fact promoted by the hybrid LDH-gallate nanomaterial. Regarding the LDH-gallate hybrid product, the cell viability also decreased when the concentration increased, and the percentage of viability at 25 µg/mL was reduced to 67% (3% error) with significant difference from 0.78 to 6.25 µg/mL with respect to the LDH matrix, giving clear proof that the combination of gallate ions with the layers of the inorganic nanoparticles increase the toxicity potential.

On the other hand, a similar toxicity effect is found with gallic, LDH and LDH-gallate when the concentration is increased to 50 µg/mL, and this result suggests that this type of nanohybrid could be effective at low concentrations. This observation is reinforced with the results at 100 µg/mL, where viability with LDH and LDH-gallate is reduced to 65% and 63%, respectively (4% error).

Conversely, the toxicity achieved by the gallic control at 50 µg/mL was 82% (7% error); even though this value is significantly different to those found with LDH and LDH-gallate (74% and 71% of viability, respectively, with 7% of error), the toxicity could be associated to the LDH matrix instead. Additionally, LDH-gallate aggregates were formed during the toxicity assays, and they could induce a different uptake and cell death mechanism than that of isolated particles [15]; thus, further assays would be needed to clarify this mechanism. Finally, it is important to mention that LDH-Gallate was capable of reducing the viability of tumor cells even when the gallate content was low (10.5%); the effectiveness could be improved if this content were increased.

## 3. Materials and Methods

### 3.1. Synthesis of LDH

The inorganic LDH matrix composed by zinc and aluminum was prepared with 2.0026 g of Zn(NO_3_)_2_∙6H_2_O and 1.0097 g of Al(NO_3_)_3_∙9H_2_O in 50 mL of distilled and decarbonated water. Thereafter, a solution made of 14% ammonia was added until pH 8 was reached. The suspension was stirred for 24 h at room temperature, then the solid was recovered by decantation, washed three times with water, and dried at 70 °C.

Intercalation of gallate ions was conducted following the anion exchange technique with three attempts: (i) one in a gallate deionized water solution adjusted to pH 7 under air atmosphere; (ii) another with a gallic acid/isopropanol solution under air atmosphere; (iii) and with a gallic acid/isopropanol solution under nitrogen atmosphere. All solutions were prepared with 0.42 g of gallic acid in 50 mL of water or isopropanol. Those solutions were mixed with 0.30 g of the LDH dried powder and stirred for 24 h. The powders were recovered by decantation, washed with water (the product prepared in water solution) or isopropanol and dried at 50 °C in a vacuum oven.

### 3.2. Nanoparticles Characterization

Infrared (IR) spectra by attenuated total reflectance were collected in a Thermo-Scientific spectrometer model Nicolet iS5 (Madison, WI, USA), with 16 scans and a resolution of 4 cm^−1^. ESR spectra were obtained in a Jeol spectrometer model FA200 operated in the X-band (~9.5 GHz) at room temperature (300 K). The powder samples were directly introduced into the quartz tube. X-ray diffraction patterns were acquired with a STONE SEIFERT diffractometer (General electric company, Schenectady, NY, USA) using Cu Kα radiation at 30 kV and 20 mA. The goniometer was rotated at a step of 0.02° with a time of 0.5 s per step. TEM analysis was conducted with a JEOL JEM1010 microscope (JEOL, Ltd., Tokyo, Japan) at100 kV.

### 3.3. Gallate Quantification and Release Experiments

The HPLC analysis was run using an Agilent Technologies chromatographer (Santa Clara, CA, USA) model 1260 Infinity, with a Thermo Scientific ODS Hypersil (Waltham, MA, USA) (150 mm × 4.6 mm, 5 µm) column operated at 25 °C. The mobile phase was composed of water (adjusted to pH 3.00 with phosphoric acid) and acetonitrile (80:20 *v*/*v*). Isocratic elution was used at a flow rate equal to 0.7 mL min^−1^ and the detection wavelength was 272 nm.

The nanoparticles sample was treated by adding 0.002 g of the powder to 1 mL of 0.10 M HCl. The mixture was stirred in a vortex apparatus for 5 min; thereafter, 0.01 mL of solution was transferred to a vial and completed the final volume of 1 mL, this solution was injected to the HPLC system.

Release of gallate from the LDH structure was monitored with 10 mg of sample added to 50 mL of 0.01 M Na_2_CO_3_ solution. The mixture was stirred at 100 rpm and 37 ± 1 °C. Aliquots of 0.5 mL were removed to quantify gallate between 30 and 1440 min. All the experiments were done in triplicate.

### 3.4. Citotoxicity Assays

Cytotoxicity studies were performed using the colorimetric methyl tetrazolium (MTT) assay. It measures mitochondrial dehydrogenase enzyme activity in living cells through the reduction of the yellow water soluble tetrazolium bromide salt-producing purple formazan, which is not soluble in water compounds [16,17]. The cell line A549 (Human lung carcinoma) was seeded in 96-well plates (Corning, New York, NY, USA) at a density of 1×10^4^ cells per well in 100 µL of Roswell Park Memorial Institute (RPMI) medium, supplemented with 10% fetal bovine serum (FBS). A 24-h period was chosen to allow cells to adhere in the 96-well plates and the cells were treated with increasing concentrations of gallate-loaded LDH nanoparticles. Pure gallic acid (Sigma-Aldrich, St. Louis, MO, USA) was used as the positive control. All the experiments were run in triplicate. After 24 h of exposure, the media were removed and each well was rinsed with PBS 1X. For each well, 100 µL of new medium prepared with 10% FBS and 10% MTT solution (5 mg/mL) (Sigma-Aldrich) was added and incubated for 4 h at 37 °C under 5% CO_2_ atmosphere, allowing for the formation of yellow formazan by viable cells. After the 4 h of incubation, the medium was discarded and 150 µL of dimethyl sulfoxide (DMSO) was added to each well in order to dissolve the dye. Absorbance was measured at 540 nm using a microplate reader. Statistical analysis to determine a significant difference between treated groups was tested by an ANOVA and Bonferroni post hoc test.

## 4. Conclusions

Applications of LDH in biomedicine are still promising. In this study, we determined that isopropanol and nitrogen atmosphere are suitable media to prepare the LDH-based nanohybrid with stable gallate ions. LDH and LDH-gallate nanoparticles are both toxic against the A549 adenocarcinoma cell line at concentrations of 50 and 100 µg/mL, while at concentrations between 0.39 and 25 µg/mL, the observed trend was a reduction of cell viability after 24 h in the LDH-gallate nanohybrid with respect to the isolated LDH and gallate compounds. Part of the toxic effect of the nanohybrid is conferred by the LDH matrix composed by zinc and aluminum cations, and this must be considered in the further design of drug vehicles based on LDH nanoparticles. Additionally, a rationalized experimental study is recommended to study the effect of aggregates formed in the culture media during bioassays as well as assays to improve the content of gallate within the LDH matrix.

## Figures and Tables

**Table 1:** Ranges of decomposition events observed in the thermogravimetric analysis (TGA) profile.

Temperature Range (°C)	Mass Loss (%)	Assignment	Reference
25–128	6	Water evaporation	[9]
128–269 and 269–343		Nitrate and hydroxyl elimination	[9]
343–448	11	Gallate decomposition	[9]
448–540	5	Decomposition of residues from the organic moiety.	[12]
540–800	1		
